# Residual effects of muscle strength and muscle power training and detraining on physical function in community-dwelling prefrail older adults: a randomized controlled trial

**DOI:** 10.1186/1471-2318-12-68

**Published:** 2012-11-07

**Authors:** Astrid Zech, Michael Drey, Ellen Freiberger, Christian Hentschke, Juergen M Bauer, Cornel C Sieber, Klaus Pfeifer

**Affiliations:** 1 Department of Movement Science, University of Hamburg, Hamburg, Germany; 2 Institute for Biomedicine of Aging, University of Erlangen-Nuremberg, Nuremberg, Germany; 3 Institute of Sport Science and Sport, University of Erlangen-Nuremberg, Erlangen, Germany; 4 Geriatric Center Oldenburg, Klinikum Oldenburg, Oldenburg, Germany

**Keywords:** Prefrailty, Exercise, Mobility, Residual effects, Detraining

## Abstract

**Background:**

Although resistance exercise interventions have been shown to be beneficial in prefrail or frail older adults it remains unclear whether there are residual effects when the training is followed by a period of detraining. The aim of this study was to establish the sustainability of a muscle power or muscle strength training effect in prefrail older adults following training and detraining.

**Methods:**

69 prefrail community-dwelling older adults, aged 65–94 years were randomly assigned into three groups: muscle strength training (ST), muscle power training (PT) or controls. The exercise interventions were performed for 60 minutes, twice a week over 12 weeks. Physical function (Short Physical Performance Battery=SPPB), muscle power (sit-to-stand transfer=STS), self-reported function (SF-LLFDI) and appendicular lean mass (aLM) were measured at baseline and at 12, 24 and 36 weeks after the start of the intervention.

**Results:**

For the SPPB, significant intervention effects were found at 12 weeks in both exercise groups (ST: p = 0.0047; PT: p = 0.0043). There were no statistically significant effects at 24 and 36 weeks. In the ST group, the SPPB declined continuously after stop of exercising whereas the PT group and controls remained unchanged. No effects were found for muscle power, SF-LLFDI and aLM.

**Conclusions:**

The results showed that both intervention types are equally effective at 12 weeks but did not result in statistically significant residual effects when the training is followed by a period of detraining. The unchanged SPPB score at 24 and 36 weeks in the PT group indicates that muscle power training might be more beneficial than muscle strength training. However, more research is needed on the residual effects of both interventions. Taken the drop-out rates (PT: 33%, ST: 21%) into account, muscle power training should also be used more carefully in prefrail older adults.

**Trial registration:**

This trial has been registered with clinicaltrials.gov (NCT00783159)

## Background

Older adults are at an increased risk of deteriorating health and mobility. Mobility impairments have been shown to be associated with the occurrence of disability and are a strong predictor of mortality and nursing home admission [1]. This concerns especially the frail and prefrail cohort in the older community [2]. Frailty is commonly considered as the consequence of decline in molecular, cellular and physiological systems and describes the vulnerability of older people regarding health-related disability, dependency need for long-term care and death [3,4]. Prefrailty is defined as the transitional stage between the non-frail and frail state [5].

Considering these facts, exercise interventions aiming at enhancement in mobility and function in frail or prefrail older adults have gained increasing attention and promotion as well as scientific support. Systematic reviews [2,6,7] emphasize the potential of exercise interventions on function, mobility, fall risk, quality of life or physical activity in this population. According to Theou et al. [7] resistance, balance and multicomponent exercise interventions have a number of beneficial effects for walking speed, chair rising and stair climbing abilities as well as balance in prefrail and frail older adults. Progressive resistance training is frequently used among older adults to address muscle strength deficits, low physical performance and mobility-related issues [8,9]. The meta-analysis of Steib et al. [8] indicates a dose–response relationship of resistance training in healthy community-dwelling older adults. Greater benefits were shown for high-intensity or muscle power training programs when they were compared with moderate-intensity or traditional muscle strength training programs. In prefrail older adults however, muscle strength and muscle power interventions have been shown to be equally beneficial for increasing physical function [10]. This implies that prefrail older adults may respond differently to muscle strength or muscle power training modalities than nonfrail older adults and exercise interventions would have to be adjusted accordingly.

However, due to lack of research uncertainty exists regarding the persistence of physical adaptations in prefrail older adults following muscle strength and muscle power training. These data are of great interest since frail or prefrail older adults are generally vulnerable to disability or health-related issues [1] and might be unable to attend exercise sessions regularly. Periods without regular exercise interventions lead to a reduction in various physiological functions and can diminish positive effects of pervious exercise interventions to a certain extent [11]. Studies [11-14] that investigated the influence of detraining periods reported a progressive decline of functional capacity in older adults with a pre-training history. However, it is suggested that short-term exercise interventions effects can still be present after several months without regular physical activity.

Data on residual effects of short-term training interventions and long-term periods of detraining might help to develop future strategies for exercise programs in older adults. Thus, the objective of this study is to investigate the impact of a short-term muscle strength and muscle power training program and long-term detraining on physical function in prefrail community-dwelling older adults.

## Methods

Sixty nine prefrail community-dwelling older adults, aged 65–94 years agreed to participate in this study. The detailed description of included participants and the intervention program has previously been published [10]. Briefly, volunteers with diagnosis of depression (Geriatric Depression Scale >5) [15], dementia (Mini Mental State Examination <25) [16], BMI >35 kg/m2, intake of immuno-suppressive drugs, history of kidney stones, sarcoidosis, plasma-cytoma, chronic obstructive pulmonary disease, inflammatory bowel disease, angina pectoris, history of cancer and current participation in muscle training programs were excluded from participation. After providing informed consent, eligible older participants underwent frailty screening. According to Fried et al. [3] a phenotype of prefrailty was identified by the presence of one or two of the following five components: (a) weight loss, (b) slow walking speed, (c) low handgrip muscle strength, (d) low physical activity (Minnesota Leisure Time Physical Activity Questionnaire) [17], and (e) self-reported exhaustion (Center for Epidemiologic Studies-Depression Scale) [18].

After inclusion and before the intervention period, all participants started taking vitamin D3 orally during a 8-week run-in phase. The vitamin D3 effects were previously reported [10]. Before the start of the intervention program, participants were stratified by sex and frailty score and were randomly allocated into a muscle strength training (ST, n = 23), muscle power training (PT, n = 24) or control group (C, n = 22) by a researcher not involved in this study. Randomization was computer-generated in blocks of 12–15 participants and the blinded assessor handed out sealed envelopes with group assignment to each participant. Measurements were performed at baseline (immediately before the start of the intervention) and at 12, 24 and 36 weeks (after the start of the intervention period). The intervention period started immediately after the 8-week run-in phase with vitamin D intake. The 12 weeks of training were followed by 24 weeks of detraining, during which the participants were instructed to maintain their usual physical activity without participation in exercises intervention groups. Participants in all groups were asked to keep their physical activity level constant throughout the trial until the last measurement was performed. The study was approved by the Medical Ethics Committee of the local University and registered at clinicaltrials.gov as NCT00783159.

### Intervention

The intervention has been described previously [10] and all sessions were performed in an exercise room of the clinical setting where this study was conducted. In the first session, the participants were familiarized with the trainings devices, contraction velocity, exercises and safety measures. At the beginning of each session, the participants gave informal feedback regarding potential training-related issues or health problems. Both training groups completed a 5-min warm-up program of walking exercises, followed by 20-min of balance exercises (performed on stable ground, mats and wobble boards in combination with ball-catching exercises) and 25-min of muscle strength or muscle power exercises using the ‘Bodyspider’ resistance training machine (KOOPERA, Germany) [10]. The interventions were performed twice a week over 12 weeks. Trained instructors supervised all standardized training sessions and compliance was recorded by using exercise diaries. The PT group was instructed to move as rapidly as possible during the concentric phase of each repetition and to move slowly during the eccentric phase (approximately 2–3 s). To ensure the required movement velocity, the participants were verbally encouraged. The ST group followed the same routine, but performed the concentric and eccentric contractions with an ‘average’ velocity (2–3 s). Both intervention groups completed two sets with 2 min rest between each set. The training intensity increased continuously throughout the intervention period. Resistance was adjusted by increasing the tension of pulling forces on the resistance training machine and participants started with 15 exhausting repetitions in the first weeks (Borg’s Rate of Perceived Exertion = RPE: 10–12). The intensity increased every fortnight up to 16 RPE by reducing repetitions (6 in the final weeks) according to the guidelines of McDermott and Mernitz [19]. The exercises in both intervention groups were as follows: chest press, hip extension/flexion while standing, hip adduction/abduction while standing, tip-toe raises and chair rise.

### Outcome measures

Physical performance was measured using the summary scale of the Short Physical Performance Battery (SPPB) [1]. The SPPB scale summarizes measures of balance, gait speed and chair rise and scores between 1 (low mobility) and 12 (full mobility) points. A decreasing SPPB summary score is inversely related to an increased risk of disability, and is a strong predictor future mortality and nursing home admission [1].

Muscular power of the lower limb was tested by the sit-to-stand transfer test [20,21]. Participants were asked to rise as fast as possible from a chair into a standing position and to stand as still as possible for five seconds. During the whole measurement both feet were on a force plate (Zebris Medical, Germany) in order to determine the exact period between maximum vertical ground reaction force (start of the rising phase) and end of the rising phase.

Sit-to-stand transfer muscle power (STS-power) was calculated from the vertical force of body weight (f), the difference between height in a sitting and in an upright position (s) and the time needed for rising (t). The following equation was used: P = F·s/t [20].

The Short Form of the Late Life Function and Disability Instrument (SF-LLFDI function component, German version) [22,23] was used to assess self-reported function of lower extremity and upper extremities. Body composition was determined using a dual energy X-ray absorptiometry (DXA) scanner (Lunar Prodigy, GE Healthcare Technologies, USA). Appendicular lean mass (aLM) was calculated as the sum of the lean mass of both arms and legs. The SPPB was defined as the primary and STS muscle power, SF-LLFDI and aLM as the secondary outcomes.

### Statistical analysis

In consideration of previous authors [24,25], the SPPB score was treated as an interval-scaled variable and results are presented as mean ± standard deviation. A two factorial linear mixed model, appropriate for repeated measures data, was used to analyze continuous data in the main and secondary outcome variables. The independent continuous variables in the mixed effect model were group x time (fixed effects) and time nested in the random individual’s factor (random effect). A saturated model in a full factorial design was fitted for each considered time point. The likelihood ratio test was used as global test (all occurring time by group interactions) and t-tests for single beta coefficients. The main focus of the analysis was on the sustainability of the seen intervention effects in comparison to no training. Thus, the whole-plot factor ‘group’ with three categories (control, ST, PT) was split into two indicator variables (ST, PT) with 'control' as reference. To evaluate the sustainability of the seen direct intervention effect [10] in the main outcome variable, differences to baseline were tested in 24 and 36 week follow-ups in an a priori ordered sequence. Between-group differences in anthropometric data at baseline were assessed using t-tests. The statistical analysis was performed by a statistician using 'R' (R Foundation for Statistical Computing, Vienna, Austria).

## Results

**Figure 1:**
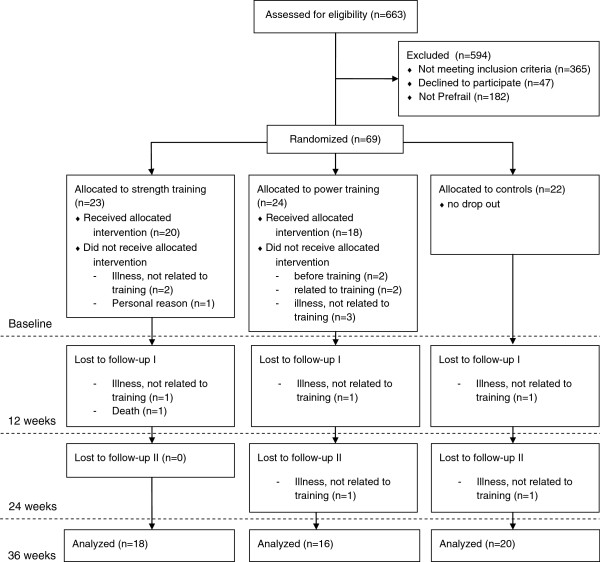
Consort diagram with participant flow.

Initially, sixty-nine participants were enrolled in this study. There were no significant differences in anthropometric data between groups at baseline, post-test as well as 24 and 36 week follow-ups (Table 1). Five participants in the ST group, eight persons in the PT group and two controls did not complete the study due to the following reasons: illness, not related to training (ST: n = 3; PT: n = 4; C: n = 2), issues related to training (PT: n = 2), no intention to start with training (PT: n = 1), personal reason interfering with commitment (PT: n = 1), poor adherence (ST: n = 1) and death (ST: n = 1). The two drop outs related to training in the PT group reported exacerbation of osteoarthritis as well as vertigo. Overall, 56 participants attended the 24 week and 54 participants the 36 week follow-up measurements (Figure 1).

**Table 1:** Characteristics of participants and primary and secondary outcomes (mean ± standard deviation)

	**Strength training group**				**Power training group**				**Controls**			
	**Baseline**	**3 Months**	**6 Months**	**9 Months**	**Baseline**	**3 Months**	**6 Months**	**9 Months**	**Baseline**	**3 Months**	**6 Months**	**9 Months**
N	23	20	18	18	24	18	17	16	22	22	21	20
Age (y)	77.8±6.1				77.4±6.2				75.9±7.8			
BMI (kg/m^2^)	29.1±4.2	28.7±4.1	28.5±4.7	28.9±4.6	28.3±4.0	27.9±4.1	30.0±5.2	30.1±5.1	28.5±4.0	28.7±4.0	28.7±6.0	29.0±5.8
Mass(kg)	78.8±10.0	78.0±10.0	76.9±9.7	77.4±9.5	74.3±10.4	72.6±10.5	78.8±12.7	79.2±12.2	75.3±13.8	75.8±13.5	74.4±13.1	75.2±12.4
SPPB (pt)	8.8±2.4	9.7±2.2*	8.9±2.3	9.1±2.2	9.0±2.1	10.1±2.3*	10.3±1.5	10.4±2.1	10.2±2.1	9.7±2.1	10.2±2.1	9.7±2.8
Balance (pt)	2.5±1.0	2.8±1.3	2.6±1.2	2.6±1.2	2.3±1.2	3.1±1.2	3.2±1.0	3.3±1.0	3.1±1.2	2.8±1.1	3.0±1.2	3.1±1.1
Gait (pt)	3.5±0.8	3.7±0.6	3.6±0.9	3.5±0.8	3.8±0.5	3.8±0.4	3.8±0.4	3.9±0.3	3.9±0.4	3.7±0.6	3.9±0.2	3.6±0.7
Chair Rise(pt)	2.8±1.2	3.3±1.0	2.6±1.1	3.0±1.2	2.9±1.1	3.2±1.1	3.3±0.8	3.2±1.2	3.2±1.0	3.1±1.2	3.2±1.2	2.9±1.3
aLM (kg)	17.9±3.3	18.0±3.3	18.3±3.5	18.6±3.4	19.2±4.4	19.1±4.2	19.4±4.4	19,7±4.4	17.1±2.6	17.5±2.6	17.5±2.6	17.6±2.7
SF-LLFDI (pt)	118.2 ±16.5	119.0 ±18.5	115.7 ±17.2	115.7 ±16.1	120.0 ±17.1	120.9 ±15.8	120.6 ±21.0	114.4 ±18.8	118.9 ±18.3	118.3 ±17.1	118.2 ±17.1	113.8 ±19.6
Power (W)	447.7 ±158.0	485.5 ±149.2	458.9 ±148.2	447.2 ±133.9	497.1 ±152.0	505.5 ±110.7	517.0 ±114.8	499.0 ±99.2	463.3 ±175.6	481.5 ±130.5	450.4 ±120.0	462.8 ±135.0

*= Significantly Different Changes over Time in Comparison to Controls (p<0.05).

aLM = appendicular lean mass; BMI = Body Mass Index; SF-LLFDI = Short form of the Late Life Function and Disability Instrument; SPPB = Short Physical Performance Battery.

The scores of the SPPB, its individual components (balance, gait, chair rise) as well as secondary outcomes are shown in Table 1. At 12 weeks, the linear mixed model revealed significant changes over time in the ST (+1.0 pt.; p = 0.005; CI 0.44, 2.58) as well as PT (+0.9 pt.; p = 0.004; CI 0.48, 2.73) groups in comparison to controls. Afterwards, the mean difference to baseline values increased slightly at 24 and 36 weeks in the PT group whereas the ST group decreased after finishing exercising (Table 2). No significant effects were found for both exercise groups at 24 weeks (ST: p = 0.87; CI −1.86, 2.18; PT: p = 0.20; CI −0.75, 3.40) as well as at 36 weeks follow-up (ST: p = 0.34; CI −1.16, 3.25; PT: p = 0.10; CI −0.37, 4.17).

**Table 2:** SPPB mean (± standard deviation) differences between baseline and post-intervention data as well as differences in means between ST /PT and controls

	**Change In SPPB from baseline**			**ST Vs. control**			**PT Vs. control**		
	**ST**	**PT**	**Controls**	**Effect (Beta coefficient)**	**P Value**	**CI**	**Effect (Beta coefficient)**	**P Value**	**CI**
At 12 Weeks	1.0±1.9*	1.0±1.5*	−0.5±1.9	1.51	0.005	0.44, 2.58	1.61	0.004	0.48, 2.73
At 24 Weeks	0.0±3.5	1.9±2.4	0.3±3.4	0.16	0.87	−1.86, 2.18	1.32	0.20	−0.75, 3.40
At 36 Weeks	−0.2±3.9	1.8±3.0	−0.5±4.1	1.05	0.34	−1.16, 3.25	1.90	0.10	−0.37, 4.17

*= Significantly Different Changes over Time in Comparison to Controls (p<0.05).

SPPB = Short Physical Performance Battery; ST = Strength Training; PT = Power Training.

The individual components of the SPPB differed in their changes over time. Mean balance and chair rise scores increased following PT and kept unchanged until week 36 whereas ST resulted in an initial increase and decreased afterwards. No or minor changes over time were shown for the gait score in both intervention groups (Table 1).

There were no intervention effects at 12, 24 and 36 weeks in SF-LLFDI, aLM and sit-to-stand transfer muscle power. Muscle power increased slightly following ST but there were no changes over time in all three groups at 24 and 36 weeks.

## Discussion

The results showed that both interventions improved the SPPB score at 12 weeks in prefrail community-dwelling older adults but did not lead to statistically significant residual effects when the training is followed by a period of detraining. After stopping regular exercising, the SPPB declined continuously over time in the muscle strength training group and was at baseline level at 36 weeks follow-up. In the PT group however, the immediate intervention effect did not diminish at follow-ups. The lack of significance of these effects might be explained with the low sample size in this group at 24 and 36 weeks and thus, with a low statistical power (resulting from a high drop-out rate). However, the absolute difference between baseline SPPB and both follow-ups can be considered clinically relevant in this population since it has been shown that a substantial meaningful change in the SPPB score ranges between 0.99 and 1.34 points [25]. This implies that muscle power training might be more beneficial than traditional muscle strength training for residual effects after exercise stop. It can be also suggested that an intervention period of three months could be sufficient for persisting functional improvements in prefrail older adults.

Influences of different training intensities on detraining effects in older adults with an increased fall risk have previously been shown by Hauer et al. [14]. They investigated the effects of a high-intensity training program comprising muscle strength, functional and balance exercises in comparison to a low-intensity exercise program in community-dwelling geriatric patients with a history of injurious falls. Although the immediate high-intensity training effects declined with increasing time of detraining, the differences between the groups in most functional performances were still significant two years later.

When looking at the individual components of the SPPB, it has been shown, that the finding in the the main outcome is predominantly reflected by the balance and chair rise scores whereas the gait score remained nearly unchanged over time. The immediate effects in the balance and chair rise components can be explained with the combined use of resistance as well as balance exercises in this study. Interestingly, ongoing effects in the PT group were not only shown in the muscle strength (chair rise) but also in the balance component. This implies that the functional improvement following muscle power training was also induced by an increased standing stability. In a meta-analysis, Steib and colleagues [8] compared the effects of muscle power training and progressive muscle strength training in adults aged 65 years or more. Based on their findings, muscle power training is suggested to be more effective for enhancing functional performance in older adults than progressive muscle strength training. They reported that chair rise and stair climbing abilities improved more with muscle power training. Despite the similarity in effectiveness of both interventions after 12 weeks in our study, the present findings on the sustainability of SPPB improvements seem to support the superior effects of muscle power training shown by Steib et al. [8].

Mixed data were shown in previous studies on effects of longer periods without exercising after muscle strength or muscle power training. Henwood and Taaffe [11] reported a decline in dynamic and isometric muscle strength as well as muscle power following detraining in healthy older adults, which were previously involved in regular muscle strength and muscle power training programs. They also found residual functional ability effects which were comparable between both training modalities. Another trial [12] reported no influence of different muscle strength training intensities (light, moderate and high) on detraining-induced changes in muscle strength in healthy older adults. This implies that in healthy older adults, in relation to muscle strength the training modality or intensity may have no influence on potential detraining effects. However, significantly different detraining adaptations in peak and mean muscle power were shown between low-intensity and high-intensity muscle strength training groups for inactive older men [13]. Following 8 months of detraining in the low-intensity training group, muscle power declined whereas at the same follow-up measurement there were still significant training effects in the high-intensity training group. Consequently, it is suggested that changes due to longer periods without regular training following previous exercise interventions may depend on the physical precondition or activity status of the study population.

Contrary to other studies in this field however, no effects were found on STS muscle power as well as aLM. Especially the lack of muscle power effects by using the sit-to-stand transfer test in this study does not reflect the shown changes in the chair rise component of the SPPB. For the STS muscle power test, the participants were asked to rise as fast as possible from a chair into a standing position and to stand as still as possible for five seconds on a force plate whereas the SPPB included 5 quickly repeated chair stands. In both tests, the time needed to complete the task is used for data analysis. Thus, it might be possible that the chair rise test of the SPPB with 5 repeated stands is more sensitive to training-induced changes than the STS transfer test with only one stand. Furthermore, it is also likely that the prefrail participants in this study might have been responded differently to training than other study populations.

The absence of significant effects on aLM reflects the findings of previously published studies that reported only small or no training-induced changes of lean body mass in older adults [26-28]. This can be most probably explained with neurological adaptations resulting in an increased voluntary activation [29] as well as rapid motor unit activation and higher firing rates [30].

There are several issues that might have limited the generalizability of our findings and should be considered for practical use. In this study, we used a resistance-training machine with elastic bands. Resistance was adjusted by increasing the tension of pulling forces of the elastic bands. Besides the chair rise, all lower extremity exercises were performed in a standing position. Often, the participants performed single-leg exercises during which they had to stabilize on the non-exercising leg. The increased instability during exercising might have led to inadequate perceptions of exertion and thus, inadequate muscle strength and muscle power training intensities. It is also likely that the high-velocity contractions in the PT group were more challenging for keeping balance than the low-velocity muscle strength exercises. This would also explain the predominant changes in the balance component of the SPPB in the PT group.

Furthermore, during the intervention period the drop-out rate was more than twice as high in the muscle power training group than in the muscle strength training group. In two participants in the muscle power training group, the drop-out was directly related to the exercise intervention. This indicates that the physical demands and perceived exertion of muscle power exercises are exceptionally high and may increase the risk for adverse events in the prefrail population. The explicit use of prefrail community-dwelling older adults in this study is another issue concerning the limited generalizability of our findings. Studies regarding residual effects of muscle power or muscle strength training reported mixed findings [11-13] suggesting that the physical performance status may play a major role in the duration of physical adaptations during detraining.

## Conclusions

In conclusion, the present study showed that muscle power and strength training in prefrail older adults did not statistically differ in their sustainability of intervention effects when the training is followed by a period of detraining. The SPPB declined continuously over time in the muscle strength training group but remained nearly unchanged in the muscle power training group at 24 and 36 weeks follow-ups. This indicates maintenance of effects in prefrail older adults after finishing the muscle power intervention program. However, in consideration of the overall and training-related drop-out rate in this study, muscle power training in prefrail older adults should be prescribed with care and must be monitored by specially trained exercise instructors. For optimized exercise prescriptions in prefrail older adults, future studies are needed comparing immediate and residual effects as well as potential adverse events of different training intensities, durations, frequencies and type of programs.

## Abbreviations

aLM: Appendicular lean mass; BMI: Body Mass Index; DXA: Dual energy X-ray absorptiometry; GDS: Geriatric Depression Scale; MMSE: Mini-Mental State Examination; PT: Muscle power training; SF-LLFDI: Self-reported function of the Late Life Function and Disability Instrument; SPPB: Short Physical Performance Battery; ST: Muscle strength training; STS: Sit-to-stand transfer.

## Competing interests

There are no conflicts of interest to report.

## Authors’ contributions

Study concept and design: AZ, MD, EF, JB, CS, KP; Acquisition of data: AZ, MD; Statistical analysis: CH; Interpretation of data: AZ, MD, EF, JB, CS, KP; Drafting of the manuscript: AZ; Critical revision of the manuscript: AZ, MD, EF, CH, JB, CS, KP. All authors read and approved the final manuscript.

## Pre-publication history

The pre-publication history for this paper can be accessed here:

http://www.biomedcentral.com/1471-2318/12/68/prepub
